# Antivirals against Monkeypox (Mpox) in Humans: An Updated Narrative Review

**DOI:** 10.3390/life13101969

**Published:** 2023-09-26

**Authors:** Giuseppe Bruno, Giovanni Battista Buccoliero

**Affiliations:** Infectious Diseases Unit, San Giuseppe Moscati Hospital, Azienda Sanitaria Locale Taranto, 74121 Taranto, Italy; giovannibattis.buccoliero@asl.taranto.it

**Keywords:** monkeypox, Mpox, antivirals, tecovirimat, brincidofovir, cidofovir

## Abstract

As of 29 August 2023, a total of 89,596 confirmed cases of Mpox (monkeypox) have been documented across 114 countries worldwide, with 157 reported fatalities. The Mpox outbreak that transpired in 2022 predominantly affected young men who have sex with men (MSM). While most cases exhibited a mild clinical course, individuals with compromised immune systems, particularly those living with HIV infection and possessing a CD4 count below 200 cells/mm^3^, experienced a more severe clinical trajectory marked by heightened morbidity and mortality. The approach to managing Mpox is primarily symptomatic and supportive. However, in instances characterized by severe or complicated manifestations, the utilization of antiviral medications becomes necessary. Despite tecovirimat’s lack of official approval by the FDA for treating Mpox in humans, a wealth of positive clinical experiences exists, pending the outcomes of ongoing clinical trials. Brincidofovir and cidofovir have also been administered in select cases due to the unavailability of tecovirimat. Within the scope of this narrative review, our objective was to delve into the clinical attributes of Mpox and explore observational studies that shed light on the utilization of these antiviral agents.

## 1. Introduction

Beginning in May 2022, there was an unprecedent global spread of monkeypox (Mpox), so much so that on 23 July of the same year, the Mpox outbreak was declared a Public Health Emergency of International Concern (PHEIC) by the World Health Organization (WHO) [1]. Until that time, documented cases of Mpox were primarily observed in central and western Africa, where outbreaks were periodically reported [2,3]. According to the WHO, between 1 January 2022, and 29 August 2023, there have been 89,596 confirmed cases of Mpox, including 157 fatalities, reported in 114 countries worldwide, with a significant reduction in reported cases in recent months since the peak in August 2022 [4]. 

Mpox is a double-stranded DNA virus belonging to the Poxviridae family, orthopoxvirus genus (the same genus as the Variola virus that causes smallpox and the Vaccinia virus that causes cowpox), firstly isolated in a laboratory in Denmark in 1958 [5]. Mpox was initially observed in humans in 1970 within the Congo, subsequently manifesting in outbreaks within rural areas of countries in central and western Africa [6,7]. It emerged as a significant orthopoxvirus concern for public health following the eradication of smallpox in 1980 [8]. The two genetic clades of the virus are Clade I (previously known as the Congo Basin clade) and Clade II (the former west African clade), which was responsible for the Mpox outbreak 2022. The latter is characterized by a more insidious clinical course [9]. In 2003, the first outbreak beyond Africa was reported in the United States, succeeded by occasional cases documented in the United Kingdom, Israel, and Singapore [10]. Mpox is transmitted via viral zoonosis. In endemic countries, transmission predominantly occurs through contact with animals that are mostly asymptomatic and serve as natural reservoirs [11]. However, the primary transmission mode documented in the most recent Mpox outbreak involved direct human-to-human contact with infectious skin or lesions, including those in the oral or genital areas [12]. This encompasses various forms of contact such as skin-to-skin (touching or genital/anal contact), mouth-to-mouth (kissing), mouth-to-skin (oral–genital contact or kissing on the skin), as well as face-to-face transmission through respiratory droplets (talking or breathing) or short-range aerosols resulting from prolonged close contact. Additional modes of transmission have been recorded, including transplacental and perinatal transmission [13]. The potential transmission through blood, semen, or other bodily fluids from Mpox remains a subject of debate [4,14].

As viral DNA has been detected in certain infected subjects weeks after contraction, it is advisable to use condoms for up to 8 weeks following a diagnosis of Mpox to prevent transmission [4]. While the majority of Mpox cases during the 2022 outbreak were observed in men who have sex with men (MSM), it is important to acknowledge that the infection can impact individuals of all ages and genders, as confirmed by various studies [15,16,17]. However, considering that the smallpox vaccine seems to offer approximately 85% protection against Mpox, individuals previously vaccinated against smallpox appear to be less susceptible to infection [18,19]. It is worth noting that immunity might diminish over the years due to factors like illnesses, aging, and immunosuppressive medications [20,21]. Consequently, it is crucial not to categorize Mpox solely as a disease that primarily affects the younger population.

Typically, it is a self-limiting disease, albeit with a potentially varied and occasionally subtle clinical progression [22,23]. The incubation period ranges from 1–2 days up to 21 days, and the illness can persist for up to 4 weeks or even longer [24]. Mpox is characterized by a prodromal phase lasting several days, during which systemic symptoms such as fever, general malaise, fatigue, arthromyalgia, and lymphadenomegaly emerge [25]. The latter are distinctive to Mpox in comparison to other similar illnesses. A few days after the prodromal phase, skin eruptions appear, which can involve the face and spread all over the body, sometimes also affecting the oral mucosa (in 70% of cases), genitals (30% of cases), and conjunctiva (20%) [26]. Ocular involvement can lead to corneal ulcers and blindness [27]. The skin rash typically progresses sequentially from macules (lesions with a flat base) to papules (slightly raised solid lesions), vesicles (lesions filled with transparent fluid), pustules (lesions filled with yellowish fluid), and scabs that dry up and fall off. The number of lesions varies from a few to several thousand [28]. Unlike chickenpox, the lesions are generally of the same size and at the same maturation stage at each anatomical site [29]. The clinical manifestations of Mpox in the global epidemic of 2022 exhibited distinctive characteristics, including the inconsistent presence of the prodromal phase and frequent initial involvement of the anogenital region [30]. Several complications have been described, including cutaneous abscesses, proctitis, sepsis, bronchopneumonia, keratoconjunctivitis, myocarditis, as well as systemic complications such as dehydration and malnutrition [31]. The clinical course can be more severe in immunocompromised individuals, including people living with HIV (PLWH) and in pregnant women [32,33]. 

The diagnosis of monkeypox relies on the amalgamation of anamnestic information, clinical manifestations, and laboratory analyses that corroborate the diagnosis. The polymerase chain reaction technique confirms the diagnosis [34,35]. Typically, several skin swabs are extracted from the affected areas. The differential diagnosis is frequently challenging, given that other infectious conditions like chickenpox, herpes simplex, or syphilis can induce similar lesions [36]. Lymphadenopathy in the prodromal phase allows the differentiation between Mpox and chickenpox [37]. 

In the majority of cases, it is a self-resolving illness that does not require any treatment [38]. Occasionally, supportive therapy is needed, especially in situations where dehydration has occurred along with alterations in kidney function and/or bacterial complications [39]. However, certain conditions exist in which antiviral treatment can prove immensely valuable, and at times, even life-saving. Immunocompromised individuals, including those with suppressed immune systems such as PLWH, may experience a more subtle disease progression characterized by multiple localized lesions that can give rise to complications [40]. Consequently, during the 2022 outbreak, antiviral medications that had previously demonstrated efficacy against orthopoxviruses were employed [41]. Notably, tecovirimat, brincidofovir, and cidofovir were among the agents used. The purpose of this narrative review is to provide an overview of the most recent insights pertaining to the main antiviral treatments against Mpox. This entails delineating their pharmacokinetic and pharmacodynamic attributes, along with detailing the findings from a range of clinical studies, including retrospective and prospective analyses, case reports, and randomized trials.

## 2. Therapeutic Options against MPX

A literature search was conducted across key scientific databases, including PubMed, EMBASE, and SCOPUS, utilizing the following terms: “Monkeypox”, “MPX”, “Mpox”, “MPX therapy”, “Monkeypox treatment”, “antivirals MPX”, “tecovirimat”, “brincidofovir”, and “cidofovir”. The selection of studies cited in this narrative review was carried out at the authors’ discretion, prioritizing papers with larger sample sizes, more recent publication dates, preferably available in full text and written in English. We considered 29 August 2023 as the most recent date for the literature research conducted. Given that this is not a systematic review but a narrative review, we did not apply the PRISMA guidelines.

**Figure 1:**
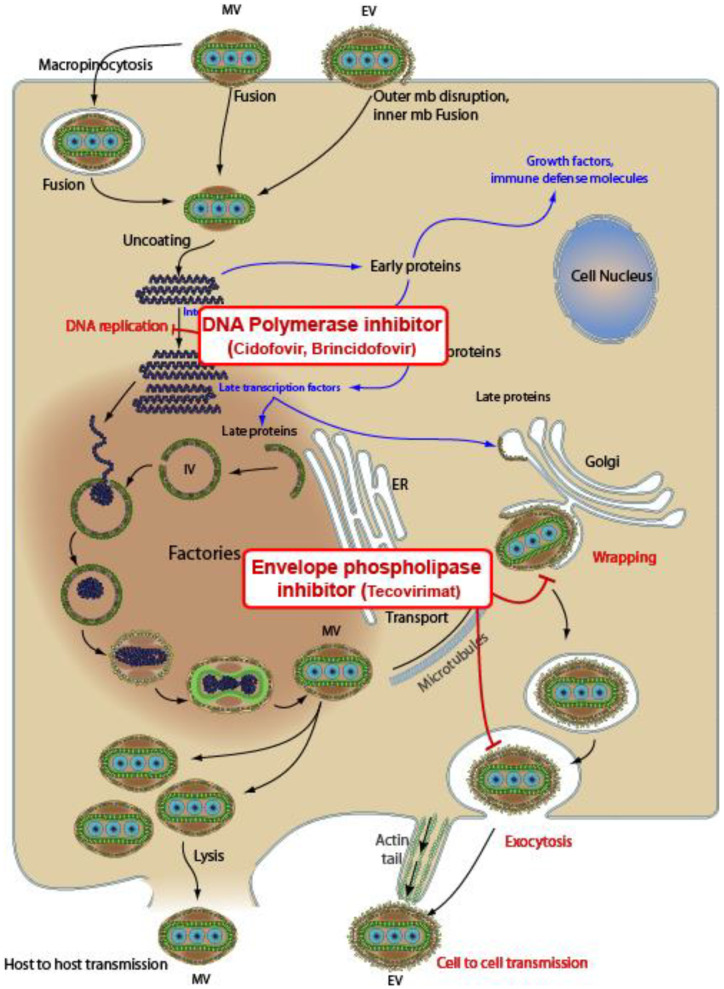
Mechanisms of action of three antivirals against Mpox. Tecovirimat inhibits the VP37 protein, which plays a crucial role in envelope formation. Cidofovir and brincidofovir (a prodrug of cidofovir) are DNA polymerase inhibitors. Sources: CDC Smallpox Prevention and Treatment; downloaded from https://viralzone.expasy.org/9958 (accessed on 29 August 2023). The picture is under creative commons 4.0 license.

While several compounds appear to exhibit in vitro activity against orthopoxviruses [41,42,43], our review exclusively concentrated on three antiviral agents for which there is a more substantial body of literature during the last Mpox outbreaks in 2022: namely, tecovirimat, brincidofovir, and cidofovir. The role of Vaccinia Immune Globulin Intravenous (VIGIV) and recommended or investigational vaccines extends beyond the scope of this narrative review’s purpose. In any case, we have added a brief discussion of the main available vaccines, which serve as a valuable tool capable of containing the spread of Mpox. In the subsequent Table 1, the primary pharmacokinetic and pharmacodynamic characteristics are outlined. A dedicated section has been crafted for each antiviral, referencing pertinent studies. In Figure 1, the mechanisms of action of the three antiviral agents against Mpox are illustrated.

## 3. Tecovirimat

Tecovirimat (ST-246, TPOXX) is a small molecule that exhibits antiviral activity against smallpox, cowpox, and monkeypox [44]. It hampers extracellular viral proliferation by inhibiting the VP37 protein, which plays a pivotal role in envelope formation [45]. 

Tecovirimat received approval from the US FDA in 2018 for the treatment of smallpox, even though this disease was declared eradicated in 1980 [46,47]. The effectiveness of tecovirimat against Mpox had been substantiated through several preclinical studies [48,49,50]. In these animal-based investigations, tecovirimat exhibited the capacity to significantly lower mortality rates among animals exposed to Mpox, achieving survival rates of no less than 90% [51]. Nevertheless, when treatment commencement was delayed, its efficacy in averting mortality exhibited a reduction. 

In Europe, as of January 2022, this antiviral is the sole treatment authorized by the European Medicines Agency (EMA) for Mpox [52]. It is suitable for use in both adults and children weighing a minimum of 13 kg. It is available in an oral formulation, which is preferred, or intravenous for those who have difficulty swallowing.

Since tecovirimat has not yet been approved by the FDA for use against Mpox, healthcare providers are encouraged to enroll individuals with confirmed Mpox in the phase 3 randomized controlled clinical trial NCT05534984 called STOMP (Study of Tecovirimat for Human Monkeypox Virus) [53]. This trial aims to assess the safety and efficacy of tecovirimat in treating this illness. Furthermore, access to oral tecovirimat treatment is also available for individuals with monkeypox who meet the eligibility criteria through the CDC’s Expanded-Access Investigational New Drug (EA-IND) protocol [53,54]. Tecovirimat use should be considered in individuals with the following conditions:

- Severe disease (hemorrhagic disease; large number of lesions such that they are confluent; sepsis; encephalitis; ocular or periorbital infections; or other conditions requiring hospitalization);
- Involvement of anatomic areas that might result in serious sequelae including scarring or strictures and severe infections (including secondary bacterial skin infections), especially those that require surgical intervention, such as debridement.

Tecovirimat should also be considered for use in people who are at high risk for severe disease including:

− People currently experiencing severe immunocompromise due to conditions such as: HIV/AIDS leukemia, lymphoma, generalized malignancy, solid organ transplantation, therapy with alkylating agents, antimetabolites, radiation, tumor necrosis factor inhibitors, or high-dose corticosteroids, being a recipient of a hematopoietic stem cell transplant <24 months post-transplant or ≥24 months but with graft-versus-host disease or disease relapse, or having autoimmune disease with immunodeficiency as a clinical component;
− Pediatric populations, particularly patients younger than 8 years of age;
− Pregnancy or breastfeeding women;
− People with the following conditions: atopic dermatitis, eczema, burns, impetigo, varicella zoster virus infection, herpes simplex virus infection, severe acne, severe diaper dermatitis with extensive areas of denuded skin, psoriasis, or Darier disease (keratosis follicularis).

While the results of clinical trials are not yet accessible, the literature contains some observational studies and case reports that have documented the safety and efficacy of this antiviral in severe or complicated cases of Mpox, particularly among immunocompromised individuals [55]. Recent real-life studies reported a favorable outcome and good safety profile in individuals with severe Mpox [56,57,58,59], as described in Table 2. With the exception of a few studies, the majority of these are primarily case series, and their results should be interpreted and contextualized while awaiting the results of randomized trials, which will provide more robust and less contentious data. A recent investigation aimed to evaluate the effect of the use of tecovirimat in subjects with confirmed Mpox. This study included hospitalized individuals with Mpox, of whom 19 were treated with tecovirimat and 22 were untreated subjects [60]. The authors did not notice an advantage in terms of reducing viral replication and achieving clinical recovery five days after treatment commencement. They concluded that it is imperative to await the outcomes of randomized trials, suggesting that the use of the antiviral could potentially prove beneficial if administered early after symptom onset, possibly during clinical trials.

A recent study compared the safety profiles and clinical outcomes in a population of 196 individuals with or without HIV infection treated with tecovirimat [61]. Both groups exhibited similar rates of hospitalization, treatment indications, and concurrent infections. No significant differences were observed in treatment outcomes, including the time to improvement or the rate of persistent symptoms. The authors concluded that among the patients treated with tecovirimat for severe Mpox, the presence of HIV did not seem to have an impact on treatment outcomes.

Conversely, notable data arose from a multicenter study that assessed the clinical presentations and results of 382 PLWH who had Mpox [62]. Among them, 228 individuals (65%) were adherent to antiretroviral therapy (ART), 193 (51%) out of 382 had an undetectable viral load, and 32 (8%) had a concurrent opportunistic illness. Overall, 107 (28%) of 382 were hospitalized, of whom 27 (25%) died. Among the 27 individuals who died, 10 people had completed one or two full courses of tecovirimat. Of note, individuals with severe immunosuppression, such as those showing a CD4 count below 200 cells/mm^3^, experienced a more aggressive clinical course with a higher risk of complications including the necrotizing forms, prolonged hospitalization, and unfavorable outcomes. The authors proposed that severe immunosuppression and immune reconstitution following the initiation of antiretroviral therapy (ART) were linked to the most notable complications observed. The role of antivirals against Mpox in such severe cases is still to be defined, taking into consideration that a prolonged treatment can also result in drug resistance, as reported in three subjects in this study and described in other studies. [63,64].

Considering the role of tecovirimat in individuals with immunosuppression beyond HIV infection, additional pertinent information comes from the study by Higgins et al., which assessed the use of this antiviral in solid organ transplant recipients, primarily kidney, with confirmed Mpox [65]. In this setting, the authors found that the rates of hospitalization were high (73%, n = 8) with a median length of stay of 4.5 days (range 1–10 days) and one death observed. 

Recently, a Cochrane Review was undertaken to evaluate therapies targeting Mpox, consisting of two primary components: a thorough evaluation of evidence extracted from randomized controlled trials (RCTs) and a descriptive analysis of safety data obtained from non-randomized studies [66]. No evidence from randomized controlled trials was identified by the authors regarding the efficacy and safety of therapeutic interventions for individuals with Mpox. On a different note, the review of non-randomized studies indicated very low-certainty evidence suggesting the absence of significant safety concerns associated with the application of tecovirimat in individuals afflicted with MPX infection. The authors conclude that further advancements and outcomes from clinical trials could offer more substantial evidence regarding the efficacy of antiviral agents against MPX, thus paving the way for a future update of this Cochrane Review.

**Table 2:** Clinical studies evaluating tecovirimat as a therapeutic option against Mpox.

Author Study, Year and Reference	*n.*	Type of Study	*n.* of PLWH	*n.* Treated with Tecovirimat	Control Group	Clinical Setting	Outcome	Notes
Thornill, 2022 [55]	528	Retrospective	41%	2%	No	Outpatients and inpatients	70 (13%) were hospitalized. No deaths were reported.	Mild clinical course. Only 13% of the persons were admitted to a hospital with a low rate of non-serious complications.
Hermanussen, 2023 [56]	12	Case series	8	All	No	11/12 hospitalized	All subjects with severe or complicated MPX. No deaths.	Treatment with tecovirimat was well tolerated and all individuals showed clinical improvement.
Raccagni, 2023 [57]	9	Case series	2	All	No	3 hospitalized	Complete resolution of symptoms after a median of 12 days; clinical improvement after 3 days from the prescription.	Treatment well tolerated without severe adverse events.
						6 outpatients		
Desai, 2022 [58]	25	Compassionate use	9	All	No	Outpatients and inpatients	Complete resolution of lesions was reported in 10 patients (40%) on day 7 of therapy, while 23 (92%) had resolution of lesions and pain by day 21. No deaths.	Minimal adverse events.
								Conclusions related to antiviral use vs. natural evolution of disease should be made with caution
Matias, 2022 [59]	3	Case series	1	All	No	Hospitalized	No severe MPX or complications. No deaths.	A mild increase in ALT in one patient resolved without tecovirimat discontinuation
Mazzotta, 2023 [60]	42	Case series	15	19	Yes	All hospitalized	Among the 41 patients included, 19 completed a course of tecovirimat. The median time from symptom onset to hospitalization and drug initiation was 4 days and 10 days, respectively.	The authors found no evidence for a significant effect of tecovirimat in shortening healing time and viral clearance.
Mc Lean, 2023 [61]	154	Retrospective cohort study	72	All	No	16 hospitalized	Groups had similar rates of hospitalization, indications for treatment, and co-occurring infections, but PWH had fewer days from symptom onset to treatment (7.5 vs. 10).	Four participants had serious adverse events; none were attributed to tecovirimat. Twenty-two percent of participants had non-severe adverse effects. HIV status did not seem to affect treatment outcomes.
Mitià, 2023 [62]	382	Retrospective study	All	62 (16%)	No	107 (28%) of 382 were hospitalized, of whom 27 (25%) died.	107 (28%) of 382 were hospitalized, of whom 27 (25%) died. Among the 27 individuals who died, 10 people had completed one or two full courses of tecovirimat.	All deaths occurred in people with CD4 counts of less than 200 cells per mm^3^. Three individuals had laboratory confirmation of tecovirimat resistance.
Higgins, 2023 [65]	11	Case series	NA	All	No	11 subjects with a history of organ transplantation. The majority were kidney transplant recipients (91%, *n* = 10).	Eight were hospitalized during the clinical course. There was one Mpox-related death in the cohort. Infection was reported to have resolved at 30-day follow-up in all other cases.	Median duration of symptoms at presentation was 6 days (range 3–14 days). Rates of hospitalization were high (73%, *n* = 8) with a median length of stay of 4.5 days (range 1–10 days).

Abbreviations: NA, not available; PLWH, people living with HIV.

## 4. Brincidofovir

Brincidofovir, sold under the brand name Tembexa, is an antiviral employed for the treatment of smallpox [67]. Functioning as a prodrug of cidofovir, brincidofovir is chemically designed in such a manner that it becomes associated with a lipid molecule. This design enables the compound to release cidofovir within cells, leading to elevated concentrations of cidofovir within cells and reduced levels in the bloodstream [68]. The drug undergoes cleavage to yield cidofovir, which is further phosphorylated to create cidofovir diphosphate, an active metabolite. This metabolite, in a subsequent step, thwarts DNA polymerization through competitive inhibition with deoxycytosine-5-triphosphate (dCTP) for viral DNA polymerase. This disruption eventually culminates in the inhibition of viral replication [69]. 

In contrast to cidofovir, brincidofovir offers the benefit of inducing fewer adverse effects, including nephrotoxicity, which has been noted in instances of intravenous administration in both animals and humans [70]. Another advantage compared to cidofovir is its oral administration in the form of tablets or a suspension, which is beneficial for individuals with swallowing difficulties. Brincidofovir demonstrated activity against numerous DNA viruses including cytomegalovirus (CMV) and adenoviruses with a specific focus on poxviruses such as Mpox [71]. 

After tecovirimat, brincidofovir is the second drug to have received formal FDA approval for use against smallpox in June 2021 [72,73]. However, the effectiveness of brincidofovir in treating smallpox has not been ascertained in humans due to the impracticality of conducting thorough and well-controlled field trials. Brincidofovir is made available through the SNS (Strategic National Stockpile) for the treatment of Mpox to medical practitioners who request and obtain an FDA-authorized, single-patient, emergency-use Investigational New Drug (e-IND) [53]. Brincidofovir can be considered for use under an e-IND in the treatment of human monkeypox disease in both adults and pediatric patients, including neonates, who meet the following criteria [53]:

− Having severe disease or at a high risk of progressing to severe disease, and fulfilling either of the following conditions:
  (1) Showing clinically significant disease worsening while on tecovirimat treatment, or experiencing disease recurrence (initial improvement followed by a deterioration) after an initial period of improvement on tecovirimat.
  (2) Being otherwise ineligible for or having a medical reason preventing the use of oral or intravenous tecovirimat.

Of note, clinical data supporting the use of brincidofovir in the treatment of Mpox are lacking and primarily stem from small case series or individual case reports [62,74], as described in Table 3. Currently, the off-label utilization of brincidofovir in real-world scenarios has not yielded compelling data to support its routine use against Mpox. Brincidofovir did not demonstrate an effective clinical benefit, as reported in a study involving three patients [74]. Furthermore, the use of Brincidofovir is linked to an elevation in liver enzymes, which can be concerning [75]. For this reason, during its use, the meticulous monitoring of hepatic function is mandatory. Further insights will be gained from randomized clinical trials, such as NCT01143181 [53].

## 5. Cidofovir

Cidofovir stands as a broad-spectrum antiviral, displaying efficacy in addressing a diverse array of viruses [76]. This spectrum includes herpes viruses (HSV-1 and HSV-2), varicella zoster virus (VZV), CMV, Epstein–Barr virus (EBV), papovaviruses (of the Papovaviridae family) such as Papillomavirus (HPV) and Polyomavirus, adenoviruses (of the Adenoviridae family), and poxviruses (of the Poxviridae family) [77]. Cidofovir is a nucleotide analogue of cytidine monophosphate. It functions by selectively inhibiting the synthesis of viral DNA [78]. It achieves this by inhibiting the viral DNA polymerase and also competing with the enzyme’s natural substrate. Cidofovir obtained FDA approval in 1996 for treating CMV retinitis in patients with AIDS and is commercially available in an injectable form [79]. Certain in vitro and animal studies have demonstrated some degree of effectiveness against poxviruses [80]. The unavailability of both tecovirimat and brincidofovir in many Western countries during the 2022 Mpox outbreak led some clinicians to employ off-label cidofovir, particularly for severe or complicated forms of Mpox, yielding some promising outcomes [81], as described in Table 4. In fact, a case series originating from the San Raffaele Scientific Institute in Milan, Italy, detailed the cases of four males afflicted with severe Mpox [82]. Each of these individuals received a single intravenous dose of cidofovir between the months of June and August in the year 2022. The authors of these reports observed a rapid amelioration within a matter of days in all cases. This was evident through a reduction in the quantity of lesions and the formation of crust, alongside the resolution of initial symptoms. Consequently, there was no necessity for additional cidofovir administrations. Notably, no adverse events or emergence of new symptoms were reported following the administration of cidofovir in these cases. We presented a case involving a young man who had a complicated form of Mpox and had been recently diagnosed with AIDS. Additionally, his immune system had shown limited signs of recovery [83]. Similar to other reports where two doses or more were required [84,85], this individual was successfully treated by administering two doses of cidofovir with a one-week interval between them. Other positive clinical experiences involved the topical application of cidofovir to treat Mpox lesions [86]. The limited availability of robust studies investigating the use of cidofovir in Mpox underscores the necessity for comprehensive research endeavors to evaluate factors such as effectiveness, optimal dosage, timing, and administration route.

## 6. Vaccines against Mpox: A Brief Summary

The objective of the worldwide multi-country outbreak response for Mpox is to halt the transmission from one person to another, giving special attention to communities that face a high risk of exposure, which can vary depending on the specific circumstances. The strategy also involves implementing robust public health measures to effectively prevent the disease from spreading further [4]. The careful administration of vaccines can play a crucial role in supporting this response. Currently, there are three vaccines that have been considered and approved in various regions for use during Mpox outbreaks [87]. These three vaccines, initially developed to fight smallpox, are ACAM2000, MVABN (also known as Imvamune, JYNNEOS, or Imvanex), and LC16. Their effectiveness against Mpox is under ongoing evaluation. Several randomized clinical trials are currently underway to assess their short-term and long-term protective efficacy. MVA-BN is a non-replicating vaccine administered in two subcutaneous doses, spaced at least 4 weeks apart. In contrast, LC16 and ACAM2000 are minimally replicating and replicating vaccinia-based vaccines, respectively. They are administered as a single dose using the scarification method. According to the WHO, mass vaccination against Mpox is not currently recommended. Instead, primary preventive vaccination is advised for high-risk groups, including individuals who have multiple sexual partners and healthcare workers. As for other categories at risk of developing severe forms, such as immunocompromised individuals, pregnant women, and children, vaccination should be provided, but only when there is a concrete risk of exposure. In the absence of immunocompromised conditions, all three vaccines are suitable options. However, individuals with significant immune system impairments, such as transplant recipients or cancer patients, should exclusively receive the MVA-BN vaccine. This recommendation also applies to pregnant and breastfeeding individuals for both primary and post-exposure preventive vaccination.

## 7. Future Perspectives

The global emergence of Mpox in 2022 has, at last, shifted focus towards this previously overlooked and less understood illness, extending awareness beyond the boundaries of countries where it was endemic. This has also emphasized the urgency of developing randomized and controlled clinical studies to assess the safety and effectiveness of antiviral compounds. The conclusive results from these trials will provide the necessary evidence to ensure swift and optimal treatments, enabling timely containment of its spread and better-equipped responses to potential future outbreaks with stronger scientific backing. 

Meanwhile, research efforts persist in the pursuit of identifying both established and novel molecules capable of inhibiting viral entry or the replication of orthopoxviruses [41]. Among these, there are certain drugs already used for tumor-related conditions, such as imatinib and mitoxantrone, as well as antibiotics like rifampicin. Through distinct mechanisms, these substances can hinder viral synthesis and replication [88,89,90]. Nevertheless, clinical efficacy data for these products against Mpox in humans are lacking. Other promising molecules exhibit antiviral properties by targeting specific molecular sites. An example of such molecules are silver-included nanoparticles, which have demonstrated effective antimicrobial and antiviral attributes against a variety of organisms [91]. Rogers et al. have explored the utilization of silver nanoparticles, both polysaccharide-coated and non-coated, with varying diameters, as well as silver nitrate at different concentrations, as inhibitors for Mpox infectivity [92]. Other viral inhibition mechanisms are represented by RNA interference in which the expression of target genes is inhibited by the attack of exogenous genes [93,94,95].

## 8. Conclusions

The lack of consistent and robust data from both observational studies and randomized controlled clinical trials prevents the scientific community from reaching definitive conclusions about the effectiveness of antiviral agents with activity against Mpox. During the Mpox outbreak of 2022, observational studies, including case series and case reports, nonetheless provided encouraging data regarding the safety and effectiveness of tecovirimat. It is plausible that the use of antiviral agents could be particularly beneficial during the early stages of the infection, before the disease spreads and the lesions increase in number and extent. Therefore, in cases where it is indicated and in specified situations such as severe and/or complicated forms, it is essential not to delay antiviral treatment, preferably administered within the framework of clinical trials. The evolving landscape of the literature regarding the use of antiviral treatments, including tecovirimat, in the context of Mpox, is expected to be of significant interest and is likely to grow in the near future. The results of randomized trials are expected to provide crucial data that will contribute to establishing a scientific evidence base. These findings will help guide informed decisions in addressing this illness effectively. The early recognition of the disease, the proper and judicious use of antiviral treatments, vaccination in at-risk populations or on a larger scale in areas where Mpox is endemic, educational and risk prevention efforts, along with healthcare policies, could collectively contribute effectively to countering the widespread dissemination of Mpox.

## Figures and Tables

**Table 1:** Pharmacological features of the three antivirals with activity against Mpox.

	Tecovirimat	Brincidofovir	Cidofovir
MPX EC50	0.07–0.16 µM	0.07–1.2 µM	27–78 µM
Mechanism of action	inhibits viral protein p37, which is involved in the final steps of maturation of the virus, thus reducing the production of extracellular virus	DNA polymerase inhibitor	DNA polymerase inhibitor
T 1/2	18–26 h	19.3 h (CDV diphosphate 113 h)	3.2–4.4 h (intracellular t1/2 significantly longer)
Protein binding	77–82%	>99.9%	<6%
Elimination	73% urine (predominantly as metabolites); 23% feces (predominantly as parent drug)	Excreted in urine and bile as metabolites	The primary route of elimination is renal, with approximately 90% of the total dose cleared by the kidneys
Pharmaceutical preparation	200 mg capsules	100 mg tablets; 10 mg/mL oral suspension	Injection
	Injection (10 mg/mL)		
Mode of administration	Oral or IV	PO only	IV
Dosage	Oral (take within 30 min of full meal): 13–24 kg: 200 mg Q12h; 25–39 kg: 400 mg Q12h; 40–119 kg: 600 mg Q12h; 120 kg or above: 600 mg Q8h	<10 kg: 6 mg/kg (suspension) once weekly × 2 doses (day 1 and 8); 10 kg to <48 kg: 4 mg/kg (suspension) once weekly × 2 doses (day 1 and 8); 48 kg and above: 200 mg (20 mL or 1 tablet) once weekly × 2 doses (day 1 and 8); Tablets: ≥48 kg: 200 mg on days 1 and 8 (no food required)	5 mg/kg IV once a week × 2 weeks (may repeat 5 mg/kg every other week thereafter); no definitive dosing data for poxviruses. Properly timed IV prehydratation with normal saline and probenecid: 2 g po 3 h before each dose and further 1 g doses 2 and 8 h after the cidofovir infusion.
	Injection: 3–34 kg: 6 mg/kg Q12h over 6 h; 35–119 kg: 200 mg Q12h over 6 h; 120 kg and above: 300 mg Q12h over 6 h		
Duration of treatment	14 days	2 doses (day 1 and 8)	Data limited; Mpox model gave 5 mg/kg as a single dose
FDA approval	Adults and children weighing at least 3 kg for treatment of human smallpox	Adult, pediatric, and neonates for treatment of human smallpox	Treatment of CMV retinitis in patients with AIDS
Renal dose adjustment	No dosage adjustment required for capsules; contraindicated as injection if CrCl < 30 mL/min	No dosage adjustment required	Contraindicated if CrCl ≤ 55 mL/min
Hepatic dose adjustment	No dose adjustment	No dosage adjustment required	No data
Use in Pregnancy	No human data; safe in animals	May cause fetal harm based on animal data. No human data available	Not recommended in pregnancy
Most common adverse events	Headache, nausea, abdominal pain, and vomiting	Diarrhea, increased transaminases (2–7%) or bilirubin, vomiting. May irreversibly impair fertility in animal studies	Neutropenia, decreased ocular pressure, nephrotoxicity, and dose-dependent tubular injury (Fanconi-like syndrome); probenecid: hypersensitivity reactions, rash, nausea, and vomiting

Abbreviations: BID, bis in die (twice daily); CDV, cidofovir; CMV, cytomegalovirus; CrCl, creatinine clearance; EC50, half-maximal effective concentration; FDA, Food and Drug Administration; IV, intravenous; PO, per os (by mouth); Q8h, every 8 h; Q12h, every 12 h; t1/2, half-life.

**Table 3:** Clinical studies evaluating brincidofovir as a therapeutic option against Mpox.

Author Study, Year and Reference	*n.*	Type of Study	*n.* of PLWH	*n*. Treated with Brincidofovir	Control Group	Clinical Setting	Outcome	Notes
Mitjà, 2023 [62]	382	Retrospective study	All	7 subjects (2%) treated with brincidofovir or cidofovir	No	PLWH with advanced HIV infection	Overall, 107 (28%) of 382 were hospitalized of whom 27 (25%) died.	Severe complications were more common in people with a CD4 cell count of less than 100 cells per mm³.
Adler, 2022 [74]	7	Retrospective study	None	3	No	Hospitalized	All subjects underwent full recovery.	All subjects experienced a reversible elevation of transaminases.

Abbreviations: PLWH, people living with HIV.

**Table 4:** Clinical studies evaluating cidofovir as a therapeutic option against Mpox.

Author Study, Year and Reference	*n.*	Type of Study	*n.* of PLWH	*n.* Treated with Cidofovir	Control Group	Clinical Setting	Outcome	Notes
Mitjà, 2023 [62]	382	Retrospective study	All	2% (brincidofovir or cidofovir)	No	PLWH with advanced HIV infection	Overall, 107 (28%) of 382 were hospitalized, of whom 27 (25%) died.	Severe complications were more common in people with a CD4 cell count of less than 100 cells per mm³
Mondi, 2023 [81]	19	Case series	7	4	No	Hospitalized	Complete recovery was observed in all patients with a median of 15 days from treatment start.	Cidofovir was well tolerated.
								No significative alterations of blood tests were observed, apart
								from a transient increase in alanine aminotransferase after cidofovir.
Raccagni, 2023 [82]	4	Case series	2	4	No	Hospitalized	The authors reported a rapid improvement within days in all cases, evidenced by a decrease in numbers and crusting of Mpox lesions, and resolution of presenting symptoms.	Further administrations of cidofovir were not required. No reported adverse events or any new symptoms were observed after cidofovir administration.
Fabrizio, 2023 [83]	1	Case report	1	1	No	Hospitalized	The patient underwent full recovery, despite his immune system showing limited signs of recovery due to a recent diagnosis of AIDS.	This individual was successfully treated by administering two doses of cidofovir with a one-week interval between them.
Stafford, 2023 [84]	1	Case report	1	1	No	Hospitalized	He was discharged 52 days after his second admission and is well on follow-up.	Subject with prolonged and severe illness. A clinical response to cidofovir was evidenced, despite a previous full course of tecovirimat; he received two further doses of cidofovir, one at day seven and another at day 21 after the first dose.
Moschese, 2022 [85]	4	Case series	1	1 (a subject who was HIV negative)	No	Hospitalized	Full recovery and discharge after 8 days.	The subject received two doses of cidofovir 5 mg/kg at days 1 and 7

Abbreviations: PLWH, people living with HIV.

## Data Availability

Data is available upon request from the corresponding authors.
